# Effect of Treatment of Neuropathic and Ischemic Diabetic Foot Ulcers with the Use of Local Ozone Therapy Procedures—An Observational Single Center Study

**DOI:** 10.3390/clinpract14050169

**Published:** 2024-10-16

**Authors:** Jarosław Pasek, Sebastian Szajkowski, Grzegorz Cieślar

**Affiliations:** 1 Collegium Medicum im dr Władysława Biegańskiego, Jan Długosz University in Częstochowa, 13/15 Armii Krajowej St., 42-200 Częstochowa, Poland; 2 Faculty of Medical and Social Sciences, Warsaw Medical Academy of Applied Sciences, 8 Rydygiera St., 01-793 Warszawa, Poland; sebastianszajkowski@wp.pl; 3 Department of Internal Medicine, Angiology and Physical Medicine, Faculty of Medical Sciences in Zabrze, Medical University of Silesia in Katowice, 15 Stefana Batorego St., 41-902 Bytom, Poland; cieslar1@o2.pl

**Keywords:** etiology of diabetic ulcers, healing time, chronic wounds, pain ailments, local ozone therapy

## Abstract

**Background:** Diabetes ranks high among worldwide global health problems, and diabetic foot ulcer syndrome (DFU) is considered as one of its most serious complications. The purpose of this study was to evaluate the impact of local ozone therapy procedures on the wound healing process in patients with two DFU types: neuropathic and ischemic. **Material and Methods:** In the retrospective study reported here, the treatment outcomes of 90 patients were analyzed: 44 males (48.8%) and 46 females (51.2%), in the age range between 38 and 87 years of age, with neuropathic (group 1) and ischemic (group 2) diabetic foot ulcers treated by means of local ozone therapy. The assessment of therapeutic effects in both groups of patients included an analysis of the rate of ulcer healing using planimetry and an analysis of the intensity of pain associated with ulcers performed using the VAS scale. **Results:** After the application of ozone therapy procedures, a statistically significant decrease in the surface area of the ulcers was obtained in both groups of patients, respectively: in group 1 from 7 (6–7.5) cm^2^ to 3 (2–3.5) cm^2^ and in group 2 from 7.5 (6.5–8) cm^2^ to 5 (4.5–5.5) cm^2^ (*p* < 0.001), with a complete healing of ulcers not observed in any patients from groups 1 and 2. After treatment, the surface area of the assessed ulcers was smaller in the neuropathic group. The intensity of pain experienced after treatment also decreased with statistical significance in both groups (*p* < 0.001). **Conclusions:** Short-term local ozone therapy was effective in promoting wound healing and alleviating pain in patients with DFUs of both neuropathic and ischemic etiology. The effectiveness of therapy in the neuropathic type of DFUs was significantly higher than in the ischemic type, in which patients had a higher incidence of risk factors and more advanced lesions, characterized by a larger initial ulcer area and greater intensity of pain.

## 1. Introduction

In the 21st century, diabetes mellitus (DM) has become a global epidemic. According to the International Diabetes Federation, approximately 425 million people worldwide currently suffer from diabetes, which entails 8.8% of adults aged 20–79, of whom 212.4 million are unaware of being affected by the disease. If current epidemiological trends continue, 693 million people will have developed diabetes in the world by the year 2045 [1,2].

There are numerous chronic complications in the course of DM, with one of its most serious complications being diabetic foot ulcers (DFUs). It is estimated that the incidence of DFUs worldwide affects 6.3% of the population, with the percentage as high as 7.2% in Europe, and the estimated lifetime incidence of DFUs in diabetic patients amounting to 15% [3,4].

In Poland, ulcers that develop in the course of diabetic foot ulcer syndrome, affecting approximately 10% of diabetic patients, result from damage to the skin of the feet (inadequate foot care, injuries), which is facilitated by such comorbidities as: diabetic neuropathy, occurring in approximately 28–40% of patients, and peripheral artery disease (PAD), observed in 25% of patients. In the event of a DFU and its incorrect treatment, delayed healing (usually resulting from accompanying bacterial infections) may lead to amputation of the foot or entire lower limb, causing serious clinical, physical and social–psychological consequences, as well as significant economic costs of rehabilitation. A serious problem arises from the fact that approximately 16% of patients require a further amputation within a year after the first procedure. Both organ complications of diabetes and comorbidities that co-occur with DFUs increase the mortality rate in patients with this disease. Therefore, prevention, education of patients and their families, and a multidisciplinary approach to therapy all play an extremely important role; in particular, the appropriate treatment of infectious complications in diabetic foot ulcer syndrome is essential here, which may reduce the number of amputations in the range of 49–85% [5,6,7].

The clinical division of diabetic foot ulcer syndrome is based on its etiology. There are three types of disorders: neuropathic, ischemic, and diabetic foot ulcers, which each affect the disability of patients to varying degrees [8].

As it turns out, the most common form is the neuropathic diabetic foot ulcer, which occurs in 45–60% of patients. In the processes by which hyperglycemia causes damage to peripheral nerves (motor and sensory ones), oxidative stress, adenosine triphosphate deficiency, the activity of protein kinase C, pro-inflammatory processes, and the polyol pathway are all involved. Reduced or completely lost pain ailments, temperature, vibration, and the absence of tactile sensation are observed in the foot, which may contribute to the patient’s lack of response to factors damaging the foot tissues. In conditions of hyperglycemia, inhibition of the production of endothelial nitric oxide causes the development of microcirculation disorders (increased number of arterio–venous connections), increased inflammation, and abnormal growth of the vascular intima. Among these diabetic neuropathies, the motor, sensory, and autonomic ones can be distinguished. The concomitance of these forms of neuropathy causes the development of skin hemorrhages and wounds, and ultimately leads to deformation of the feet, resulting in the loss of the supporting functions of the limb [8,9].

A diabetic foot ulcer of ischemic etiology most often develops due to coexisting PAD and occurs in approximately 15–20% of diabetic patients. Hyperglycemia causes changes in peripheral vessels, which result in endothelial cell dysfunction and reduced secretion of vasodilators, leading to limb ischemia [9]. Chronic ischemia causes symptoms of intermittent claudication in the form of pain in the calf area that appears during physical exertion—mainly walking, which, in the initial phase, disappears after a short rest. If proper treatment is not introduced early enough, after a few years, in approximately 10–20% of patients, a mild form of ischemia—intermittent claudication turns into severe ischemia with constant pain at rest, which intensifies at night [9,10].

The interplay of the patho-physiological factors discussed above favors the development of a mixed form of a diabetic foot ulcer, i.e., a neuropathic–ischemic one, which occurs in approximately 25–30% of patients with peripheral neuropathy and coexisting atherosclerotic vascular lesions. Patients with this form of diabetic foot ulcer most often experience chronic pain, especially at night when lying on their back, which disappears after lifting the affected limb [9,10].

Prevention and early detection of DFUs within the framework of guideline-based multidisciplinary care are crucial in order to reduce the morbidity and consequences of DFUs [11]. Treatment of diabetic foot ulcer syndrome (especially chronic ulcers) requires a broad spectrum of actions [12]. The basic elements of therapy include the metabolic control of diabetes, the use of appropriate footwear (comfortable with orthopedic insoles, especially in the case of foot deformities), the use of appropriate dressings depending on the local condition of the ulcers, as well as other unconventional methods of treatment (vacuum assisted closure—VAC and platelet rich plasma—PRP methods, larval therapy, hyperbaric oxygen treatment, ozone therapy, magnetic field treatment) [13,14,15]. Depending on the patient’s health, physical activity is recommended in each case, contributing to improved blood circulation. Antibiotic treatment should be introduced only after bacteriological examination (culture of the wound swab and antibiogram). In some cases, local surgical procedures are necessary (cleaning of necrotic tissue, drainage of abscesses), as well as the consultation of a vascular surgeon in cases of advanced atherosclerosis (qualification for revascularization, angioplasty, or vascular bypass procedures). In many cases, the treatment of diabetic foot ulcers requires long and intensive care, which affects the patient’s quality of life and all associated costs of chronic health care. Amputations and mortality in DFUs are complications that occur late in the course of the disease progression and are usually the result of incorrect treatment.

So far, efforts to improve the care of patients with DFUs have not led to any systematic decline in the rate of amputations, which indicates deepening difficulties in access to organized diabetes care [11,16,17].

Ozone is a gas existing in nature, which can also be produced by medical generators. Ozone is one of the most effective oxidants that easily dissolves in the water of either plasma, skin, or extracellular fluids; moreover, depending on its concentration and form of application, it can be protective or harmful for humans [18].

Medical ozone can be used for therapeutic purposes both in parenteral and topical forms or in loco–regional forms. Topical applications that can be useful in the treatment of DFUs are usually conducted by isolating skin lesions with bags or cups inert to ozone and by insufflating the mixture with ca. 95% gaseous oxygen and ca. 5% gaseous ozone or with ozonated water and/or oil [18].

During exposure of human tissue to a gas mixture composed of oxygen and ozone in therapeutic doses, both gases dissolve in the tissue depending on their solubility, partial pressure, and temperature. These gases generate lipid oxidation products and reactive oxygen species (ROS), including hydrogen peroxide. This reaction, considered as acute, mild, controlled, or transitory oxidative stress, is essential to biological stimulation of processes supporting the regeneration of DFUs, including vasodilatation caused by increased release of nitric oxide, nitrosothiols, and autacoids, promoting angiogenesis and a disinfectant action on most pathogens, as well as a release of some growth factors from platelets and endothelial cells which induce the healing of necrotic ulcers [18,19,20,21,22,23,24]. 

According to the World Federation of Ozone Therapy—WFOT, results of clinical studies have indicated that ozone therapy is often successfully applied in the treatment of many diseases of first category, such as osteomyelitis, empyema pleural, abscesses with fistulae, infected wounds, ulcers by pressure, chronic ulcers, burns, and DFUs [18].

In recent years, several papers confirmed the therapeutic efficacy of various forms of ozone therapy in the treatment of DFUs [16,25,26,27,28]. 

The purpose of this study was to evaluate the impact of local ozone therapy procedures on the wound healing process in patients with two DFU types—neuropathic and ischemic.

## 2. Material and Methods

The study was conducted in accordance with the Declaration of Helsinki (1964), and its protocol has been approved by the Local Bioethical Committee of the Medical University of Silesia in Katowice, Poland (approval reference number: KNW/0022/KB1/102/II/16/19, dated 2 July 2019). All patients enrolled in the study provided signed written informed consent approval for all procedures realized in the study.

In this retrospective study, the treatment results of 90 patients with DFUs in the course of type 2 diabetes hospitalized in the Department of Internal Medicine, Angiology, and Physical Medicine in Bytom (Poland) from January 2021 to May 2024 were analyzed. The patients ranged in age between 38 and 87 years (average age 64.4 ± 10.3 years; median 66; min 38; max 87).

Each of the analyzed patients was treated in the clinic for a period of 2.5 months. During hospitalization, all patients received identical standardized pharmacological treatment (sulodexide, micronized purified flavonoid fraction, pentoxifylline, and acetylsalicylic acid in standard doses) in order to maintain proper fasting serum glucose levels below 6 mmol/l. Procedures for cleansing wounds of necrotic tissue, daily wound inspection, specialized dressings, and local ozone therapy using the Ato-3 device produced by Metrum Cryoflex (Blizne Łaszczyńskiego, Poland) were all part of the ozone therapy protocol. Topical application of a gas mixture composed of 5% gaseous ozone and 95% gaseous oxygen (with a concentration of 40 µg/mL insufflated into an “Ozone bag” isolating a patient’s leg with skin lesions), with close monitoring of the patient, was conducted once a day for 5 days a week, with a weekend (Saturday–Sunday) break. Each therapeutic procedure lasted 30 min. The procedures were conducted in two cycles consisting of 15 procedures each (for a total of 10 weeks). The intermission between both series of procedures was 4 weeks. It was introduced in order to avoid the potential risk of the negative effects of long-term exposure to ozone on the skin around the ulcer and in profound tissues.

Inclusion criteria for the study were the following: patients of both sexes with type 2 diabetes, age range 18–90 years, with diagnosed DFUs (neuropathic or ischemic type) Wagner grade 2 or higher, in whom ulcers have not healed for at least 1 month or have recurred, and who have not undergone vascular intervention for medical reasons, with no contraindications for the use of ozone therapy treatments, and who gave their consent for treatment with the use of local ozone therapy. The following exclusion criteria were applied: patients aged <18 and >90 years, type 1 diabetes, DFUs of mixed etiology (neuroischemic), previous surgical interventions, autoimmune diseases and malignant tumors, and the absence of complete clinical data.

The patients were assigned to two research groups which differed in the type of diabetic foot ulcer: group 1 included 39 patients with neuropathic ulcers (19 female and 20 male ones) and group 2 included 51 patients with ischemic ulcers (27 female and 24 male ones). A DFU with only the features of PAD was considered an ischemic ulcer. PAD was defined in cases with an ankle brachial index (ABI) value of < 0.9. Peripheral neuropathy was defined as the presence of more than one insensible area of the three sites (plantar aspect of hallux and metatarsophalangeal joint 1 and joint 5) tested per foot based on the Semmes–Weinstein 10 g monofilament. The presence of paresthesia, tingling, numbness, absence of Achilles tendon reflex, and loss of vibration sensation was also taken into account [29]. All patients were analyzed for their demographic characteristics (age, gender, BMI, ulcer duration), the presence of comorbidities (smoking, hypertension, hypercholesterolemia), the effectiveness of ulcer healing using planimetric measurements (a computer software for planimetric assessment of wound surface area was used for digital image processing) [30], and the intensity of pain assessed by means of the VAS scale.

## 3. Statistical Analysis

For statistical analysis, the Statistica 13 package (Statsoft, Kraków, Poland) was applied. In order to test the normality of data, the Shapiro–Wilk test was used. There were non-normal distributions of data. The Mann–Whitney U test and the Wilcoxon test were applied in order to compare unmatched and matched groups of non-parametric data, respectively. Qualitative variables were assessed using the chi-square test. The values of *p* < 0.05 were considered as statistically significant. The effect size was also calculated: r = 0.1 indicated a small effect; r = 0.3 indicated a medium effect; and r = 0.5 indicated a large effect.

## 4. Results

The demographic profile of patients with regard to both types of DFUs is presented in Table 1.

The average age of all subjects was 64.41 ± 10.26 years, and the average age of patients in group 1, 60.76 ± 10.79 years, was significantly lower compared with the average age of patients in group 2 (67.19 ± 8.97 years) (*p* = 0.017). 

There was no statistically significant difference between both groups regarding the gender distribution (*p* = 0.691) or the frequency of ulcer occurrence on either lower limb (*p* = 0.977).

The value of the BMI index in group 1, which was 24.69 ± 2.99 kg/m^2^ on the average, did not differ significantly compared with its average value in group 2 (25.33 ± 2.38 kg/m^2^) (*p* = 0.240).

The average duration of ulcers in group 1, which was 3.33 ± 0.71 years, was significantly lower compared with the average duration of ulcers in group 2, which was 5.16 ± 1.06 years (*p* < 0.001).

In group 1, the number of patients with hypercholesterolemia, hypertension, and smoking was statistically significantly lower compared with group 2: (9 vs. 26 patients) (*p* = 0.07), (12 vs. 28 patients) (*p* = 0.022), and (9 vs. 28 patients) (*p* = 0.002), respectively. 

The comparison of the ulcer surface area in patients from group 1 (neuropathic ulcers) with patients from group 2 (ischemic ulcers) before and after treatment is presented in Table 2. Before the start of treatment, the average value of the ulcer surface area in patients from group 1 had been median (IQR) 7 (6–7.5) cm^2^, which was statistically significantly lower compared with the average value of the ulcer surface area in group 2, which was median (IQR) 7.5 (6.5–8) cm^2^ (*p* = 0.043, r = 0.13). After the completion of therapeutic cycles performed in both groups of patients, the average value of the ulcer area decreased statistically significantly compared with the initial values before the treatment (*p* < 0.001, r = 0.88 for group 1, and r = 0.87 for group 2). The average value of the ulcer area at the end of therapy in patients from group 1 was median (IQR) 3 (2–3.5) cm^2^; this value was statistically significantly lower compared with the average value of the ulcer surface area in group 2, which was median (IQR) 5 (4.5–5.5) cm^2^ (*p* < 0.001, r = 0.78).

The comparison of pain intensity assessed with the use of the VAS scale in patients from group 1 (neuropathic ulcers) with patients from group 2 (ischemic ulcers), before the start and after the end of treatment, is presented in Table 3.

Before the start of treatment, the average intensity of pain assessed on the VAS scale (VAS score) in patients from group 1 was median (IQR) 3 (2–4) points. This score was statistically significantly lower compared with the average VAS score in group 2, which was median (IQR) 8 (7–8) points (*p* < 0.001, r = 0.87). After the treatment, the average VAS score decreased statistically significantly in both groups of patients compared with the baseline values before the start of treatment (*p* < 0.001, r = 0.89 for group 1 and r = 0.88 for group 2), with the average VAS score after therapy in patients from group 1 being median (IQR) 1 (0–2) point. This score was statistically significantly lower compared with the average value of the ulcer surface area in group 2, which was median (IQR) 3 (3–4) points (*p*< 0.001, r = 0.72).

Table 4 shows a comparison of the improvement, shown as a percentage after treatment, achieved in patients from group 1 (neuropathic ulcers) with patients from group 2 (ischemic ulcers) in terms of reducing the ulcer surface area and the intensity of pain. After the completion of therapeutic cycle, the average value of the ulcer area decreased in patients from group 1 by a median (IQR) of 57.14 (50.00–66.66)% and in patients from group 2 by a median (IQR) of 33.33 (23.07–37.50)% compared with initial values before the start of therapeutic cycle. The difference between both groups was statistically significant (*p* < 0.001, r = 0.79).

In turn then, the intensity of pain assessed with the use of the VAS scale after completion of therapeutic cycle decreased in patients in group 1 by a median (IQR) of 66.66 (50.00–100)% and in patients in group 2 by a median (IQR) of 57.14 (50.00–66.66)% compared with the initial values before the start of therapeutic cycle. The difference between both groups was statistically significant (*p* = 0.017, r = 0.25).

During the 2.5 months of treatment, a complete healing of ulcers was not observed in any patients in groups 1 and 2. A decrease in the surface area of the ulcers by more than 50% was achieved in 33 patients (84.61%) in group 1, while in patients from group 2, the decrease in the surface area of the ulcers did not exceed 50% of the initial values. In none of the patients, either in group 1 or group 2, was there any increase in the ulcer surface area after the completion of the therapeutic cycle.

After the treatment, a complete relief of pain was achieved in 12 patients (30.76%) in group 1 and in 2 patients (3.92%) in group 2. A decrease in the intensity of pain by more than 50% of the initial values was achieved in 36 patients (92.30%) in group 1 and in 41 patients (80.39%) in group 2. In none of the patients from both groups was pain aggravation after the completion of therapeutic cycle noted. During the treatment, no complications or side effects related to ozone therapy procedures were observed.

## 5. Discussion

Sufficiently early detection and proper control of diabetes at an early stage of its development can prevent the occurrence of dangerous complications, which undoubtedly include diabetic foot ulcers. Unfortunately, although many patients declare a willingness to participate in educational programs, their level of knowledge is insufficient to conduct self-monitoring and thus prevent the development of late complications of this disease, which undoubtedly includes diabetic foot ulcer syndrome [31,32].

In a systematic review by Nickinson et al., taking into account 32 references, the authors analyzed potential delays in the identification, management, and treatment of chronic and life-threatening ischemia in the case of DFUs. In all aspects assessed, studies have shown that there are time delays, which, in some cases, are significant. The causes of these problems are complex; however, they do reflect inappropriate patient behavior in seeking specialist assistance, inaccurate assessment of patients’ local condition by healthcare workers, and barriers concerning referral for specialist treatment of DFUs [33].

Marti-Carvajal et al., in turn, assessed 28 randomized clinical trials concerning DFUs in patients with type 1 or type 2 diabetes in ten countries, with 2.365 participants. The results of their analysis showed that, in all studies, the cause of foot ulcers in the course of DFUs was not precisely defined (neurological, vascular, or mixed type) [34]. 

In another work, Ince et al. assessed the course of treatment of ulcers in the course of neuropathic DFUs, looking for connections between the effectiveness of the healing process and the initial characteristics of the ulcers. The study involved 154 patients with 410 ulcers. Healing of ulcers without the need to amputate the lower limb was recorded in 91.7% of cases. The authors confirmed the close relationship existing between the duration of ulcers at the time of referral for treatment, the size of their surface, and the result of their treatment, and emphasized the importance of early specialist assessment of ulcers which developed [35].

Previous studies published between 2005 and 2021 provided many different suggestions regarding the risk factors leading to the development of DFUs. These included: age, gender, duration of diabetes, BMI value, presence of comorbidities, a high level of glycated hemoglobin, macrovascular complications, foot deformations, and inappropriate care habits [36,37,38].

This study compared the effectiveness of local ozone therapy in the two most common types of diabetic foot ulcers, differing in the pathomechanism of the development of pathological changes in the foot (neuropathic and ischemic type) in order to identify possible factors influencing the effectiveness of local ozone therapy in these patients. 

Patients with neuropathic DFUs developed diabetic foot ulcers at a younger age, which was associated with a shorter disease duration than in the case of patients with ischemic ulcers. 

Yotsu et al. also noted the occurrence of foot ulcers at a younger age in patients with neuropathic DFUs compared with patients with ischemic and neuro-ischemic DFUs and considered age as the main factor associated with healing and skin perfusion pressure (SPP) values [39]. Similar conclusions were also obtained from the results of research conducted by Miyata et al. [40].

Neither of the analyzed groups of patients differed significantly in terms of the frequency of the assessed risk factors responsible for the development of DFUs, i.e., hypercholesterolemia, hypertension, and cigarette smoking, as these factors were found significantly more often in patients with an ischemic condition than with a neuropathic diabetic foot ulcer. These observations were also confirmed by the study by Yotsu et al. [39]. Moreover, based on their own research, Wang et al. and Abeer AbdElrahman Elnour Eltilib showed that a significant connection exists between tobacco smoking and DFUs, which could be related to the stimulation of the formation of atherosclerotic plaque in blood vessels resulting from the influence of components of tobacco smoke [41,42].

In the present study, there were no differences regarding the gender distribution of both sexes, BMI values, and the frequency of surgical interventions between the study groups. Pemayun TGD et al. also reported no differences concerning gender distribution of both sexes in patients with DFUs [31]. In turn, the previously cited research by Yotsu et al. and Miyata et al. showed a higher incidence of DFUs in men than in women [29,30]. An increased risk for men of developing DFUs was also reported by Monteiro-Soares et al. [37].

It is also worth emphasizing that the surface area of the treated ulcers in both analyzed groups showed a statistically significant decrease after the treatment, and a statistically significantly greater percentage change in the size of the ulcer area was noted in patients with neuropathic DFUs.

Izadi et al., in a meta-analysis including 11 studies presenting the results of the treatment of 960 patients with DFUs, indicated that ozone therapy accelerates the healing of DFUs, reduces the amputation rates, and decreases the length of hospitalization. However, compared with standard therapies, this method does not increase the rate of complete ulcer healing. The authors suggested that further research is needed in order to enable greater homogeneity of the protocols of conducted studies and to better verify the potential beneficial effects of ozone therapy [25].

Also, in another study by Wen et al., it has been shown that ozone therapy, when applied as monotherapy or a component of combined treatment, significantly accelerates the reduction of the treated ulcer surfaces and reduces the rate of amputations in DFUs. However, no decrease in the percentage of completely healed ulcers or the duration of the hospitalization time was observed. The authors noticed a lack of adverse effects related to the ozone therapy [26].

After a bibliographical review, similar conclusions were presented by Astacio-Picado et al. that confirmed the usefulness of ozone therapy in the treatment of DFUs, taking into account its therapeutic effectiveness and safety (only a few adverse effects were observed during the ozone therapy of patients with DFUs) [27].

Sadiq et al. assessed the course of foot ulcer healing in 35 patients with the ischemic type of DFUs and in 97 patients with the neuropathic type of DFUs over a period of 2–6 months, depending on the initial level of advancement of the ulcers (determined using the Wagner classification and the Texas classification) and the stage of their infection. The authors showed that, like in the case of our study, in patients with the ischemic type of DFUs, the ulcer healing process is less effective compared with patients with the neuropathic type of DFUs and that, in both types of DFUs, the best therapeutic effects were observed in the case of superficial ulcers of stage 2 in the Wagner classification [16].

Zimny et al. also assessed the course and effectiveness of treatment of foot ulcers of neuropathic, neuro-ischemic, and ischemic etiology in 31 patients with type 1 or type 2 diabetes for over 10 weeks. The analysis of the obtained results showed that, if proper care is provided, the wound healing time in diabetic foot ulcers depends mainly on the etiological factors causing the development of the ulcer and, to a lesser extent, on its surface area [28].

Singh et al. compared the differences in the characteristics of ulcers and the ulcer healing process in patients with different types of DFUs in 42 patients (18—neuropathic type, 14—ischemic type, and 10—neuro-ischemic type). They also analyzed the age, gender, duration of diabetes, smoking, hypertension, and glycated hemoglobin (HbA1c) level, as well as the coexistence of osteomyelitis and gangrene. The results of their study confirmed that the neuropathic type of DFUs is characterized by a better response in terms of the intensity of the healing process to the treatment used compared with other types of DFUs, with the biggest number of non-healing ulcers and limb amputations occurring in patients with the ischemic type of DFUs [43].

Meloni et al. assessed the severity of clinical symptoms and ulcer surface area, as well as the results of treatment of patients with ulcers caused by DFUs in 1198 patients, and showed that patients with ischemic DFUs tend to have more severe clinical symptoms and ulcers with a relatively larger surface area, as well as inferior treatment results compared with patients with neuropathic DFUs [44].

In patients with infected wounds, including DFUs, the most important therapeutic mechanism of applied topical ozone therapy is the bactericidal effect of ozone related to the destruction by active singlet oxygen (produced during dissociation of the ozone molecule), both bacterial cell membranes (due to oxidation of the non-saturated fatty acids forming them), and enzymatic proteins located in the bacterial cytoplasm. These processes result in developing disturbances of the activity of numerous cellular organelles and lesions of desoxyribonucleic acid, leading (in consequence) to apoptosis of bacterial cells [45]. Other therapeutic mechanisms of ozone applied in the treatment of DFUs are related to reduction of the intensity of inflammation in tissues surrounding the ulcer by inhibition of the migration of mast cells, reduction of the release of some acute phase proteins and lysosomal enzymes, and stimulation of the production of eosinophils and antioxidants, as well as activation of the Krebs cycle in erythrocytes that stimulates the release of oxygen and adenosine triphosphate in tissues surrounding the ulcer. These effects enable intensification of the process of oxygenation and nutrient supply in tissues exposed to ozone [46].

In the course of DFUs, due to the development of diabetic neuropathy, sensory fibers of the nerves are damaged, with secondary atrophy or reduction in the intensity of pain sensation. Because no pain symptoms, among others, result due to the development of ischemia of the foot tissues, these symptoms may remain unnoticed by the patient for a long time, which subsequently leads to the development and progression of skin ulcers and, consequently, to the development of severe local pain [47]. This is reflected in the results of the presented study, as patients with ischemic DFUs (whose pain resulted from both the pathological effects of diabetic neuropathy and tissue ischemia) initially (before the start of treatment) experienced significantly more profound pain in the ulcer area compared with patients with the neuropathic type of DFUs; at the same time, a less profound analgesic effect of the therapy was observed.

In summarizing the literature review, Jais emphasized that, according to the vast majority of authors, for DFU treatment in specialized centers to be effective, the precise classification of a patient with an ulcer is of key importance. Classification based on the characteristic features for a specific type of DFU allows for the appropriate selection of therapeutic interventions (optimally effective in individual types of DFUs), thus increasing the probability of achieving a positive effect of therapy [8].

## 6. Limitations of the Study

Due to the small sample size, the obtained results from this single center study cannot be generalized to refer to the general population. In the study, a control group of patients and values of glycated hemoglobin are lacking, which would have allowed for a comparison of the obtained therapeutic effects with those observed in patients in which only routine therapeutic methods had been applied.

## 7. Conclusions

As it turns out, short-term local ozone therapy is effective in promoting ulcer healing and alleviating accompanying pain in patients with DFUs of both neuropathic and ischemic etiologies. The effectiveness of therapy in the neuropathic type of DFUs is significantly greater than in the ischemic type, in which patients had a higher incidence of risk factors and more advanced lesions, characterized by a larger initial area of ulceration and a greater intensity of pain.

## Figures and Tables

**Table 1:** Demographic profile of patients with regard to both groups.

	Total n (%)	Group 1 n (%)	Group 2 n (%)	* *p*
Gender				
male	44 (48.89)	20 (22.22)	24 (26.67)	0.691
female	46 (51.11)	19 (21.11)	27 (30.00)	
Age (years)				
≤65	42 (46.67)	21 (23.33)	21 (23.33)	0.232
>65	48 (53.33)	18 (20.00)	30 (33.33)	
Body Mass Index (BMI) (kg/m^2^)				
18.5–24.99	49 (54.44)	25 (27.78)	24 (26.67)	0.811
25.0–29.99	41 (45.56)	14 (15.56)	27 (30.00)	
Ulcer duration (in years)				
<6	70 (77.78)	39 (43.33)	31 (34.44)	<0.001
≥6	20 (22.22)	0 (0)	20 (22.22)	
Ulcer location				
left leg	44 (48.89)	19 (21.11)	25 (27.78)	0.977
right leg	46 (51.11)	20 (22.22)	26 (28.89)	
Hypercholesterolemia				
yes	35 (38.89)	9 (10.00)	26 (28.89)	0.007
no	55 (61.11)	30 (33.33)	25 (27.78)	
Arterial hypertension				
yes	40 (44.44)	12 (13.33)	28 (31.11)	0.022
no	50 (55.56)	27 (30.00)	23 (25.56)	
Smoking				
yes	37 (41.11)	9 (10.00)	28 (31.11)	0.002
no	53 (58.89)	30 (33.33)	23 (25.56)	
no	52 (57.78)	25 (27.78)	27 (30.00)	

* chi-square test.

**Table 2:** Comparison of the surface areas of ulcers in patients from group 1 (neuropathic ulcers) with group 2 (ischemic ulcers), before and after treatment, along with statistical analysis.

	Ulcer Surface Area (cm^2^)			** *p* _(Before vs. After)_
	Pre-Treatment Median (IQR)	Post-Treatment Median (IQR)	Effect Size	
Group 1	7 (6–7.5)	3 (2–3.5)	0.88	<0.001
Group 2	7.5 (6.5–8)	5 (4.5–5.5)	0.87	<0.001
Effect size	0.13	0.78		
* *p* _(Gr.1 vs. Gr.2)_	0.043	<0.001		

* Mann–Whitney U test; ** Wilcoxon test.

**Table 3:** Comparison of pain intensity assessed with the use of the VAS scale in patients from group 1 (neuropathic ulcers) with patients from group 2 (ischemic ulcers), before and after treatment, along with statistical analysis.

	VAS Score (Points)			** *p* _(Before vs. After)_
	Pre-Treatment Median (IQR)	Post-treatment Median (IQR)	Effect Size	
Group 1	3 (2–4)	1 (0–2)	0.89	<0.001
Group 2	8 (7–8)	3 (3–4)	0.88	<0.001
Effect size	0.87	0.72		
* *p* _(Gr.1 vs. Gr.2)_	<0.001	<0.001		

* Mann–Whitney U test; ** Wilcoxon test.

**Table 4:** Comparison of the improvement, expressed as a percentage after treatment, of patients from group 1 (neuropathic ulcers) with patients from group 2 (ischemic ulcers) in terms of reducing the size of the wound as well as reducing the pain, along with statistical analysis.

	Group 1	Group 2		* *p* _(Gr.1 vs. Gr.2)_
	Median (IQR)	Median (IQR)	Effect Size	
Percentage change of ulcer surface area post treatment	57.14 (50.00–66.66)	33.33 (23.07–37.50)	0.79	<0.001
Percentage change of VAS score post treatment	66.66 (50.00–100)	57.14 (50.00–66.66)	0.25	0.017

* Mann–Whitney U test.

## Data Availability

The datasets used and/or analyzed during the current study are available from the corresponding author upon reasonable request.

## Abbreviations

DFU	diabetic foot ulcers
DM	diabetes mellitus
PAD	peripheral artery disease
BMI	body mass index
VAS	visual analogue scale
SPP	skin perfusion pressure
VAC	vacuum assisted closure
PRP	platelet rich plasma

## References

[1] World Health Organization (2016). Global Report on Diabetes.

[2] International Diabetes Federation IDF SEA Members. https://www.idf.org/our-network/regions-members/south-east-asia/members/94-india.html.

[3] Saeedi P., Petersohn I., Salpea P., Malanda B., Karuranga S., Unwin N., Colagiuri S., Guariguata L., Motala A.A., Ogurtsova K. (2019). Global and regional diabetes prevalence estimates for 2019 and projections for 2030 and 2045: Results from the international diabetes federation diabetes atlas, 9th edition. Diabetes Res. Clin. Pract..

[4] Zhang P., Lu J., Jing Y., Tang S., Zhu D., Bi Y. (2017). Global epidemiology of diabetic foot ulceration: A systematic review and meta-analysis. Ann. Med..

[5] Araszkiewicz A., Bandurska-Stankiewicz E., Budzyński A., Cypryk K., Czech A., Czupryniak L., Drzewoski J., Dzida G., Dziedzic T., Franek E. (2017). Clinical recommendations for the management of patients with diabetes 2017. Position of the Polish Diabetes Association. Diabetol. Klin..

[6] Mrozikiewicz-Rakowska B., Jawień A., Sopata M. (2015). Organization of care for patients with diabetic foot syndrome. Guidelines of the Polish Wound Treatment Society. Wounds Treat..

[7] Schaper N., Van Netten J., Apelqvist J., Lipsky B., Bakker K. (2017). Prevention and management of foot problems in diabetes: A Summary Guidance for Daily Practice 2015, based on the IWGDF guidance documents. Diabetes Res. Clin. Pract..

[8] Jais S. (2023). Various types of wounds that diabetic patients can develop: A narrative review. Clin. Pathol..

[9] Kim J. (2023). The pathophysiology of diabetic foot: A narrative review. J. Yeungnam Med. Sci..

[10] Armstrong D.G., Boulton A.J.M., Bus S.A. (2017). Diabetic foot ulcers and their recurrence. N. Engl. J. Med..

[11] McDermott K., Fang M., Andrew J.M., Boulton E., Hicks C. (2023). Etiology, epidemiology, and disparities in the burden of diabetic foot ulcers. Diabetes Care.

[12] American Diabetes Association (2021). Summary of Revisions: Standards of Medical Care in Diabetes-2021. Diabetes Care.

[13] Margas M., Krakowiecki A. (2011). Unconventional methods of local treatment in diabetic foot syndrome. Nowa Klin..

[14] Jeffcoate W.J., Bus S.A., Game F.L., Hinchliffe R.J., Price P.E., Schaper N.C. (2016). Reporting standards of studies and papers on the prevention and management of foot ulcers in diabetes: Required details and markers of good quality. Lancet Diabetes Endocrinol..

[15] Elraiyah T., Prutsky G., Domecq J.P., Tsapas A., Nabhan M., Frykberg R.G., Firwana B., Hasan R., Prokop L.J., Murad M.H. (2016). A systematic review and meta-analysis of off-loading methods for diabetic foot ulcers. J. Vasc. Surg..

[16] Sadiq H.A., Iftikhar M., Liaqat R., Rizvi A., Hussain A., Bhatti M.I., Khan A.Z., Zafar A., Javed F. (2023). Risk of non-healing in ischemic versus neuropathic diabetic foot ulcers in relation to grade, stage of infection and treatment protocol: A follow-up study. JAIMC.

[17] Karnafel W. (2013). Infections in diabetic foot syndrome. Diabetic Foot Syndrome—Pathogenesis, Diagnostic, Clinic, Treatment.

[18] WFOT Scientific Advisory Committee (2015). WFOT’s Review on Evidence Based Ozone Therapy (Version 1).

[19] Joyner M.J., Dietz N.M. (1997). Nitric oxide and vasodilation in human limbs. J. Appl. Physiol..

[20] Kashiba M., Kasahara E., Chien K.C., Inoue M. (1999). Fates and vascular action of S-nitrosoglutathione and related compounds in the circulation. Arch. Biochem. Biophys..

[21] Aicher A., Heeschen C., Mildner-Rihm C., Urbich C., Ihling C., Technau-Ihling K., Zeiher A.M., Dimmeler S. (2003). Essential role of endothelial nitric oxide synthase for mobilization of stem and progenitor cells. Natl. Med..

[22] Stamler J.S. (2004). S-nitrosothiols in the blood: Roles, amounts, and methods of analysis. Circ. Res..

[23] Valacchi G., Bocci V. (2003). Studies on the biological effects of ozone: 11. Release of factors from human endothelial cells. Mediat. Inflamm..

[24] Valacchi G., Fortino V., Bocci V. (2005). The dual action of ozone on the skin. Br. J. Dermatol..

[25] Izadi M., Jafari-Oori M., Eftekhari Z., Jafari N.J., Maybodi M.K., Heydari S., Vahedian-Azimi A., Atkin S.L., Jamialahmadi T., Sahebkar A. (2024). Effect of ozone therapy on diabetes-related foot ulcer outcomes: A systematic review and meta-analysis. Curr. Pharm. Des..

[26] Wen Q., Liu D., Wang X., Zhang Y., Fang S., Qiu X., Chen Q. (2022). A systematic review of ozone therapy for treating chronically refractory wounds and ulcers. Int. Wound J..

[27] Astasio-Picado Á., Babiano A.Á., López-Sánchez M., Lozano R.R., Cobos-Moreno P., Gómez-Martín B. (2023). Use of ozone therapy in diabetic foot ulcers. J. Pers. Med..

[28] Zimny S., Schatz H., Pfohl M. (2002). Determinants and estimation of healing times in diabetic foot ulcers. J. Diabetes Complicat..

[29] Oyer D.S., Saxon D., Shah A. (2007). Quantitative assessment of diabetic peripheral neuropathy with use of the clanging tuning fork test. Endocr. Pract..

[30] Senejko M., Pasek J., Szajkowski S., Cieślar G., Sieroń A. (2021). Evaluation of the therapeutic efficacy of active specialistic medical dressings in the treatment of decubitus. Adv. Dermatol. Allergol..

[31] Pemayun T.G.D., Naibaho R.M. (2017). Clinical profile and outcome of diabetic foot ulcer, a view from tertiary care hospital in Semarang, Indonesia. Diabet. Foot Ankle.

[32] Mościcka P., Szewczyk M.T., Cwajda-Białasik J., Jawień A., Woda Ł. (2016). Diabetic foot ulcer as the most common complication of diabetes—Case report. Pielęg. Chir. Angiol..

[33] Nickinson A.T., Bridgwood B., Houghton J.S., Nduwayo S., Pepper C., Payne T., Bown M.J., Davies R.S., Sayers R.D. (2020). A systematic review investigating the identification, causes, and outcomes of delays in the management of chronic limb-threatening ischemia and diabetic foot ulceration. J. Vasc. Surg..

[34] Martí-Carvajal A.J., Gluud C., Nicola S., Simancas-Racines D., Reveiz L., Oliva P., Cedeño-Taborda J. (2015). Growth factors for treating diabetic foot ulcers. Cochrane Database Syst. Rev..

[35] Ince P., Game F.L., Jeffcoate W.J. (2007). Rate of healing of neuropathic ulcers of the foot in diabetes and its relationship to ulcer duration and ulcer area. Diabetes Care.

[36] Crawford F., Mccowan C., Dimitrov B., Woodburn J., Wylie G., Booth E., Leese G., Bekker H., Kleijnen J., Fahey T. (2011). The risk of foot ulceration in people with diabetes screened in community settings: Findings from a cohort study. QJM Int. J. Med..

[37] Monteiro-Soares M., Boyko E.J., Ribeiro J., Ribeiro I., Dinis-Ribeiro M. (2012). Predictive factors for diabetic foot ulceration: A systematic review. Diabetes Metab. Res. Rev..

[38] Yazdanpanah L., Shahbazian H.B., Moravej Aleali A., Jahanshahi A., Ghanbari S., Latifi S.M. (2016). Prevalence, awareness and risk factors of diabetes in Ahvaz (south west of Iran). Diabetes Metab. Syndr..

[39] Yotsu R.R., Pham N.M., Oe M., Nagase T., Sanada H., Hara H., Fukuda S., Fujitani J., Yamamoto-Honda R., Kajio H. (2014). Comparison of characteristics and healing course of diabetic foot ulcers by etiological classification: Neuropathic, ischemic and neuroischemic type. J. Diabetes Complicat..

[40] Miyata T., Yamada N., Miyachi Y. (2010). Efficacy by ulcer type and safety of lipo-PGE1 for Japanese patients with diabetic foot ulcers. J. Atheroscler. Thromb..

[41] Wang X., Yuan C.X., Xu B., Yu Z. (2022). Diabetic foot ulcers: Classification, risk factors and management. World J. Diabetes.

[42] Abeer AbdElrahman Elnour Eltilib A. (2021). Association between smoking and foot ulcer among patients with diabetes mellitus, Wad Medani, Sudan. Sudan J. Med. Sci..

[43] Singh C.G., Sil A., Sanyal D., Mandal A. (2023). Characteristics and healing of diabetic foot ulcers. J. Clin. Diagn. Res..

[44] Meloni M., Izzo V., Giurato L., Lázaro-Martínez J.L., Uccioli L. (2020). Prevalence, clinical aspects and outcomes in a large cohort of persons with diabetic foot disease: Comparison between neuropathic and ischemic ulcers. J. Clin. Med..

[45] Liu L., Zeng L., Gao L., Zeng J., Lu J. (2023). Ozone therapy for skin diseases: Cellular and molecular mechanisms. Int. Wound J..

[46] Zeng J., Lu J. (2018). Mechanisms of action involved in ozone-therapy in skin diseases. Int. Immunopharmacol..

[47] Amin N., Doupis J. (2016). Diabetic foot disease: From the evaluation of the “foot at risk” to the novel diabetic ulcer treatment modalities. World J. Diabetes.

